# Chemical Synthesis and Functional Analysis of VarvA Cyclotide

**DOI:** 10.3390/molecules23040952

**Published:** 2018-04-19

**Authors:** Claudio A. Álvarez, Paula A. Santana, Omar Luna, Constanza Cárdenas, Fernando Albericio, María S. Romero, Fanny Guzmán

**Affiliations:** 1 Laboratorio de Fisiología y Genética Marina (FIGEMA), Centro de Estudios Avanzados en Zonas Áridas (CEAZA), Coquimbo 1781421, Chile; claudio.alvarez@ceaza.cl; 2 Facultad de Ciencias del Mar, Universidad Católica del Norte, Coquimbo 1781421, Chile; msromero@ucn.cl; 3 Centro AquaPacífico, Coquimbo 1781421, Chile; 4 Núcleo de Biotecnología de Curauma, Pontificia Universidad Católica de Valparaíso, Valparaíso 2373223, Chile; paula.santana@pucv.cl (P.A.S.); omar.luna.g@gmail.com (O.L.); constanza.cardenas@pucv.cl (C.C.); 5 Department of Organic Chemistry and CIBER-BBN, Networking Centre on Bioengineering, Biomaterials and Nanomedicine, University of Barcelona, Barcelona 08007, Spain; albericio@ukzn.ac.za; 6 School of Chemistry, University of KwaZulu-Natal, Durban 4001, South Africa

**Keywords:** cyclotide, antimicrobial activity, fish pathogens, membrane damage

## Abstract

Cyclotides are circular peptides found in various plant families. A cyclized backbone, together with multiple disulfide bonds, confers the peptides’ exceptional stability against protease digestion and thermal denaturation. In addition, the features of these antimicrobial molecules make them suitable for use in animal farming, such as aquaculture. Fmoc solid phase peptide synthesis on 2-chlorotrityl chlorine (CTC) resin using the “tea-bag” approach was conducted to generate the VarvA cyclotide identified previously from *Viola arvensis*. MALDI-TOF mass spectrometry determined the correct peptide amino acid sequence and the cyclization sites-critical in this multicyclic compound. The cyclotide showed antimicrobial activity against various Gram-negative bacteria, including recurrent pathogens present in Chilean aquaculture. The highest antimicrobial activity was found to be against *Flavobacterium psychrophilum*. In addition, membrane blebbing on the bacterial surface after exposure to the cyclotide was visualized by SEM microscopy and the Sytox Green permeabilization assay showed the ability to disrupt the bacterial membrane. We postulate that this compound can be proposed for the control of fish farming infections.

## 1. Introduction

Cyclic peptides have attracted great interest in recent years due to their increased stability over linear peptides and wide range of bioactivities [1].

Most peptides show a linear structure with open ends, which makes them targets for proteolytic enzymes, thus decreasing their bioavailability [2,3]. In contrast, cyclic peptides have the unique feature that their N and C termini are joined in an amide bond to form a cyclic backbone, and they show greater stability than their linear counterparts [4,5,6,7,8]. 

Measuring 28–37 amino acid residues in length, cyclotides are the largest known family of cyclic peptides [9]. To date, more than 150 cyclotides have been characterized from plants of the *Rubiaceae*, *Violaceae*, *Cucurbitaceae*, *Solaceae*, and *Fabaceae* families [10,11,12]. The six highly conserved cysteine residues of cyclotides lead to a knotted arrangement of three disulfide bonds. An embedded ring is formed by two disulfide bonds and their connecting backbone segments, and the third disulfide bond penetrates this ring. The combination of a cysteine knot embedded in a cyclic backbone is known as a cyclic cystine knot motif (CCK). This structure confers exceptional stability against chemical, enzymatic, and thermal conditions [13]. 

Although cyclotides show complex folding, they have been obtained by solid-phase peptide synthesis (SPPS). Linear cyclotides can be chemically synthesized by standard SPPS using tert-butoxycarbonyl (Boc) or 9H-fluoren-9-ylmethoxycarbonyl (Fmoc) chemistry [14]. However, Fmoc SPPS has some advantages for automation and for synthesizing linear peptides since it involves the use of less corrosive reagents and allows simpler cleavage of the product from the resin.

Cyclotide folding is most widely achieved first by cyclization, followed by cysteine residue oxidation. Backbone cyclization is usually performed via the ‘thia-zip’ mechanism using native chemical ligation (NCL) technology, whereby a circular peptide is produced through stepwise ring expansion of thiolactone intermediates to an end-to-end thiolactone to enable lactam formation via an S-N acyl shift [15,16,17].

Moreover, entropy-driven ligation via an S-N acyl shift between the C-terminal thioester and N-terminal cysteine and enthalpy-driven cyclization based on C-terminal activation have also been explored [18]. On the other hand, the connectivity of disulfide bonds in the oxidative folding step has been studied using a range of oxidative buffers or by applying chemoselective methods [18].

Cyclotides show inherent biological properties, including neurotensin inhibitory [19], cancer cell cytotoxic [20], anti-insecticidal [21], nematocidal [22], anti-viral [23], anti-fungal [24], and bactericidal [24,25] activity. Given these properties, they have consequently attracted considerable interest from the pharmaceutical and agricultural sectors. In addition, the features of these antimicrobial molecules make them suitable for use in animal farming, such as aquaculture.

Bacterial infections in aquaculture are currently controlled almost exclusively by antibiotics, with the consequent risk of generating pathogens that are resistant to such drugs [26,27,28]. In fact, the method by which antibiotics are administered to fish in aquaculture systems means that a large part of the drugs end up becoming residues in the marine sediment, thus affecting microbiota and promoting the emergence of resistant pathogen strains [29,30,31]. In this regard, a reduced use of conventional antibiotics is considered a priority in the fish farming industry [26,27,28,32].

Here, we obtained a synthetic cyclotide VarvA, identified previously from *Viola arvensis* [33], by Fmoc chemical synthesis and cyclization based on macrolactamization through C-terminal activation. The synthetic cyclotide was analyzed for antimicrobial activity against Gram-negative bacteria that affect Chilean aquaculture. In addition, scanning electron microscopy (SEM) and Sytox Green permeabilization assays were performed in order to directly observe the response of bacterial cell morphology and membrane integrity to treatment with the cyclotide.

## 2. Results

### 2.1. Chemical Synthesis of VarvA Cyclotide

**Figure 1:**
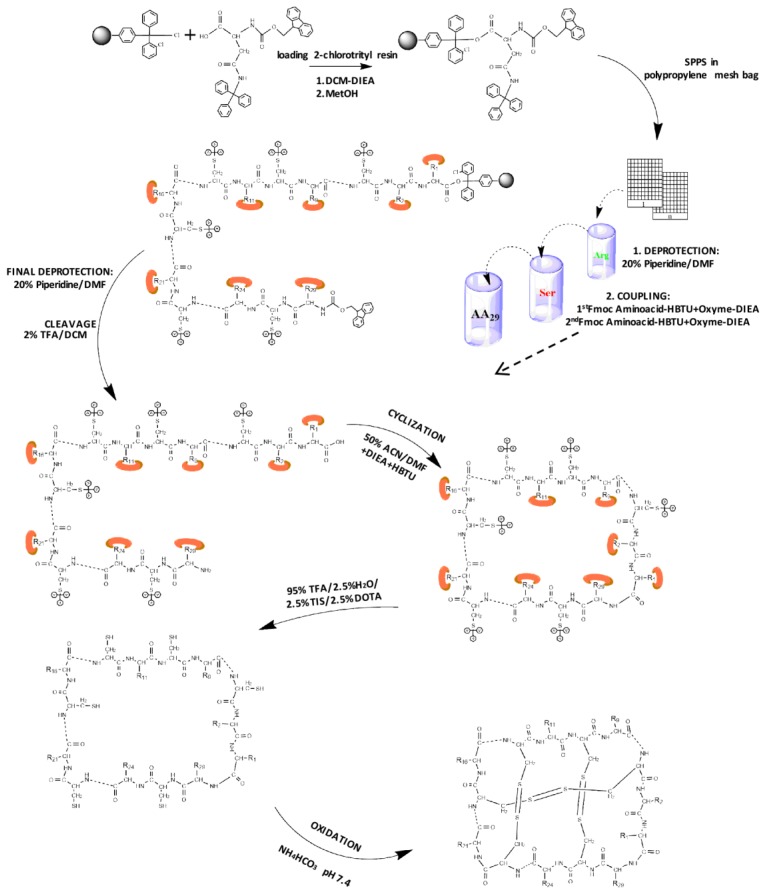
Chemical synthesis strategy. Schematic representation of synthetic route for SPPS of cyclotide.

The Fmoc-SPPS chemical synthesis approach on 2-chlorotrityl chlorine (CTC) resin was used for the VarvA cyclotide synthesis. The first Fmoc-Asn(Trt)-OH was introduced onto CTC-resin, resulting in a loading of 0.62 mmol/g resin. Next, the resin was subjected to various coupling-deprotection steps to build the linear peptide as the precursor for the cyclic peptide (Figure 1). The course of the couplings was monitored using the bromophenol test [34], which allows in situ control of the reaction since the absence of color indicates that the reaction has been completed.

The VarvA backbone (cyclo(GLPVCGECFGGTCNTPGCSCDPWPMCSRN)) was generated, starting from 2-chlorotrityl chloride resin loading with Fmoc-Asn(Trt)-OH. The second step was to place the resin in a polypropylene bag for the “tea bag” SPPS approach. Successive deprotection/coupling steps were carried out to build the peptide backbone. The peptide was then cleaved from the resin, followed by the cyclization step. Additionally, the final product yield for this step based on initial resin loading is shown. Finally, peptide oxidation was carried out to ensure correct folding and the product yield is shown. 

**Figure 2:**
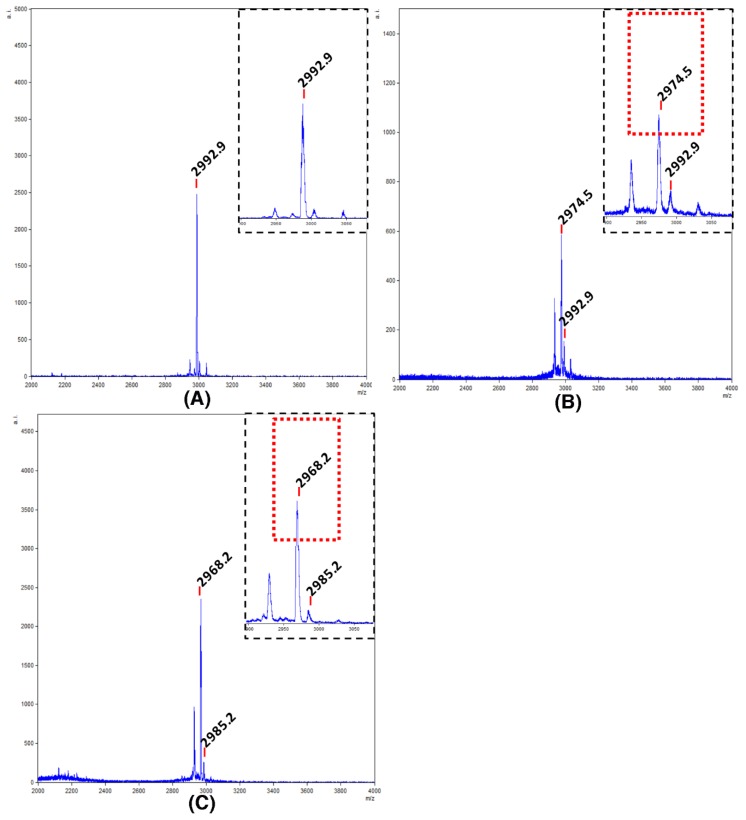
Mass spectrometric analysis of the cyclotide synthesis and folding. (**A**) MALDI-TOF spectra of linear unprotected cyclotide. Segmented quadrant shows a zoom of the molecular mass of the resulting linear peptide (2992.9 Da); (**B**) MALDI-TOF spectra of the unprotected cyclic peptide. Segmented quadrant shows a zoom of the peptide without the water molecule (2974.5 Da); (**C**) MALDI-TOF spectra of the target cyclotide. Segmented quadrant shows a zoom of the cyclic peptide without six hydrogen atoms from the three-disulfide bond formed (2968.2 Da).

Before the cleavage of the protected peptide, an aliquot of the peptide resin was treated with a high concentration of TFA to release the free peptide. The molecular mass of the resulting linear peptide (2992.0 Da) was confirmed by mass spectrometry, which showed a clean synthesis (Figure 2A). Next, a peptide concentration of 0.5 mM was used for the cyclization. In this case, the success of the cyclization was demonstrated by mass spectrometry after the global deprotection of the cyclic peptide (Figure 2B). Taking into account that ammonium bicarbonate has been successfully used for folding synthetic cyclic inhibitor peptides [35,36], we selected this buffer. The mass spectrometry analysis showed the loss of six hydrogen atoms from the three-disulfide bond formation (Figure 2C).

**Figure 3:**
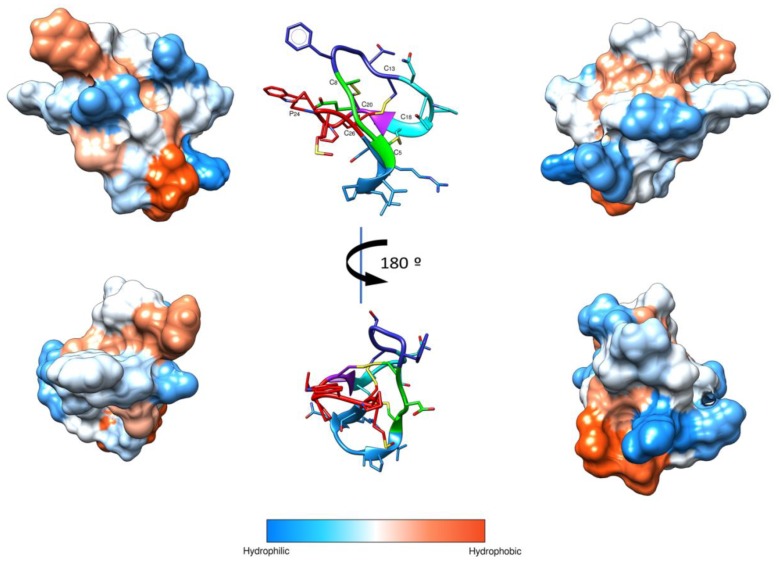
Schematic representation of VarvA structure. 3D structural model of the VarvA cyclotide in a ribbon representation is shown in the center, and disulfide bonds and P24 are indicated. The loops are colored: Loop 1 (green), Loop 2 (dark blue), Loop 3 (cyan), Loop 4 (purple), Loop 5 (red), and Loop 6 (light blue). The hydrophobic surface of the peptide was generated with a 1.4 Å probe and Kyte & Doolittle scale in UCSF Chimera [37]. Four different views of the surface are shown to facilitate visualization of the surface.

Moreover, the I-Tasser server was used to determine the three-dimensional structure. The hydrophobic surface of the structural model shows the presence of hydrophobic and hydrophilic patches (Figure 3).

### 2.2. Antimicrobial Activity of VarvA Synthetic Cyclotide against Fish Bacterial Pathogen

Here, we focused on the antimicrobial study of the VarvA synthetic cyclotide against various Gram-negative bacterial pathogens that affect fish aquaculture. The MIC concentration of synthetic cyclotide against *V. anguillarum*, *V. ordalii*, *F. psychrophilum*, *A. hydrophila*, and *A. salmonicida* was determined by a microdilution assay. The resulting MICs are shown in Table 1. According to this assay, *F. psychrophilum* was the bacteria most susceptible, with an MIC of 12.5 μM. Moreover, the peptide demonstrated good activity against *A. hydrophila* and *A. salmonicida*, with an MIC of 22.5 μM. Finally, the highest MIC was obtained against *V. anguillarum* and *V. ordalii* (30 μM).

### 2.3. Bacterial Membrane Damage Induced by VarvA Synthetic Cyclotide

**Figure 4:**
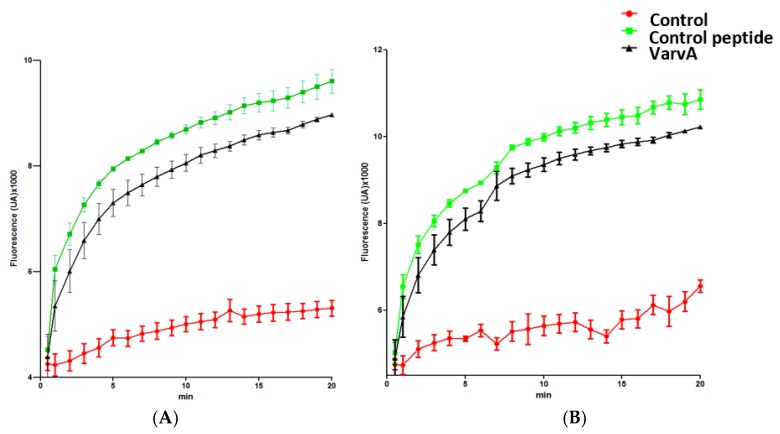
Bacterial membrane permeabilization induced by VarvA synthetic cyclotide. Membrane permeabilization influx of SYTOX Green in *E. coli* (**A**) and *F. psychrophilum* cells (**B**). The bacteria were exposed with 12.5 μM of VarvA synthetic cyclotide for 20 min in the presence of 5 µM SYTOX Green. Phospholipase-A2-derived synthetic peptide at 20 μM was used as a positive control. Negative controls were performed under the same conditions without the addition of peptide. The increase in fluorescence was recorded at 30 s intervals with the SYBR green filter.

The impact of VarvA on membrane integrity was studied by the SYTOX Green permeabilization assay. SYTOX green is selectively taken up into cells with compromised membrane integrity and exhibits greatly enhanced fluorescence upon DNA binding [38]. SYTOX green and VarvA were added simultaneously to log-phase bacteria (*E. coli* and *F. psychrophilum*), and the SYTOX Green fluorescence was quantified. Phospholipase-A2-derived synthetic peptide was used as a positive control [39]. The SYTOX green uptake was detected as early as 1 min after control peptide and VarvA treatment (Figure 4). The SYTOX green intensity increased strongly within 1 to 10 min.

**Figure 5:**
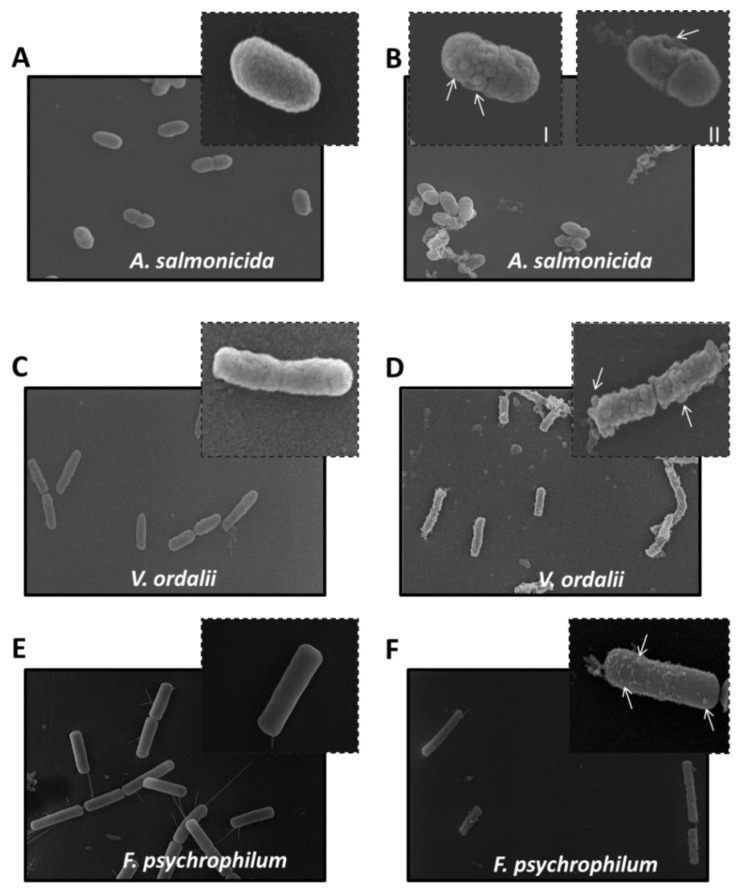
Bacterial membrane disruption induced for the synthetic cyclotide. (**A**) SEM micrographs of *A. salmonicida* subsp. *salmonicida* without peptide. Segmented quadrant shows a zoom of representative bacteria. (**B**) SEM micrographs of *A. salmonicida* subsp. *salmonicida* in the presence of 22.5 μM of synthetic cyclotide. Segmented quadrant (I) shows a zoom of representative bacteria, indicating blisters on the surface with arrows. Additionally, a bacterium with partial outer membrane detachment is shown (II). (**C**) SEM micrographs of *V. ordalii* without peptide. Segmented quadrant shows a zoom of representative bacteria. (**D**) SEM micrographs of *V. ordalii* in the presence of 30 μM of synthetic cyclotide. Segmented quadrant shows a zoom of representative bacteria indicating blisters on the surface with arrows. (**E**) SEM micrographs of *F. psychrophilum* cells without peptide. Segmented quadrant shows a zoom of representative bacteria. (**F**) SEM micrographs of *F. psychrophilum* cells in the presence of 12.5 μM of synthetic cyclotide. Segmented quadrant shows a zoom of representative bacteria indicating blisters on the surface with arrows.

In addition, SEM microscopy was used to provide direct evidence for the antimicrobial effect of VarvA synthetic cyclotide. The untreated *A. salmonicida* cells, prepared for SEM micrographs in phosphate buffer, displayed a smooth and intact surface (Figure 5A). However, after incubation with an MIC of the synthetic cyclotide, multiple blisters of various shapes were observed (Figure 5B). In addition, the partial detachment of an outer membrane was seen on *A. salmonicida* cells (Figure 5B-II). Similarly, under control conditions, the surface of *F. psychrophilum* cells and *V. ordalii* were smooth (Figure 5C,E). However, after exposure to the synthetic cyclotide, numerous blisters and partial detachment of the blisters from the membrane were observed (Figure 5D,F). 

## 3. Discussion

Cyclotides are amenable to significant sequence variation. Specifically, the backbone portions between cysteine residues, referred to as loops, can be modified [40,41]. Thus, the ability to chemically synthesize cyclotides is an important goal, both for the practical purpose of mutagenesis studies to understand their mechanism of action, as well as studies of new pharmaceutical applications. Here, we use the CTC-resin for the elongation of the peptide chain of VarvA cyclotide, an approach first described by Craik’s group [35]. The simplicity of the method was exemplified by the use of a simple tea bag as a reactor for peptide elongation. Taking advantage of the lability of the CTC-resin to acids, we used a low concentration of TFA to release the protected peptide from the resin. These conditions assure that amino acid side chains remain protected and facilitate peptide cyclization [42]. Nevertheless, for minimizing the probability of oligomerization and improving the cyclization efficiency, it is necessary to use a high dilution [43,44].

Several strategies have been included for peptide disulfide bridge arrangement, including the use of orthogonally protected cysteine residues and/or oxidation reactions promoted by reagents such as iodine, thallium trifluoroacetate, potassium ferricyanide, or dimethylsulphoxide [45,46,47,48]. However, strong oxidant compounds can also affect other amino acid residues, like tryptophan and tyrosine [36]. As an alternative, the air oxidation method performed in a buffered aqueous medium can be used. Here, we used ammonium bicarbonate for VarvA folding, supporting previous works that have used this buffer for the cyclotides disulfide bridge arrangement [49]. An additional advantage of ammonium bicarbonate is that after acidification, it is removed by lyophilization. Therefore, and although cyclotides have a difficult structure, our study demonstrates that they can be folded just by using the air oxidation method.

Cyclotides were initially studied because their main function in plants is in the control of opportunistic pathogens [50]. Given that cyclotides have an amphipathic character similar to that of classical antimicrobial peptides, it was hypothesized that they have antimicrobial activities [51]. Diverse antimicrobial properties have been described for cyclotides, including antiviral, antifungal, insecticidal, antiparasitic, and antibacterial activities [22,25,52,53,54]. Given their broad antimicrobial spectrum, cyclotides emerge as an interesting target to exploit for the improvement of animal health. 

Fish mortality caused by infectious diseases is a significant problem in aquaculture worldwide. Intensive aquaculture conditions induce fish stress, which in turn makes them susceptible to invasion by opportunistic bacterial pathogens [55]. In this regard, intensive aquaculture provides an ideal scenario in which to explore the capacity of antimicrobial peptides to protect fish against bacterial infections. Moreover, there are only a handful of approved antibiotics available and consequently the number of resistant bacteria to existing antibiotics is increasing. Here, we demonstrated the antimicrobial activity of the VarvA synthetic cyclotide against various Gram-negative bacterial pathogens that affect fish aquaculture. In addition, low MIC values were obtained for all bacteria tested. Thus, cyclotides could be of great interest even in aquaculture, as they are attractive candidates for antimicrobial therapeutic approaches.

The antibacterial properties of natural and synthetic cyclotides have been described in different works [51,56]. However, contradictory results have been reported. For example, preliminary studies showed that native kalata B1 is not active against *S. aureus*, but is active against a Gram-negative strain. Conversely, synthetic kalata B1 was found to be active against the Gram-positive *S. aureus*, but relatively inactive against Gram-negative bacteria [57]. These discordances could be explained by structural differences or by the experimental conditions used [25]. Here, we used SEM microscopy to provide direct evidence for the antibacterial effect of the synthetic cyclotide. The SEM micrographs showed that untreated bacteria displayed a smooth and intact surface, but after incubation with VarvA, multiple blisters and partial membrane detachment were observed. This membrane disruption was supported by the SYTOX Green permeabilization assay. Taken together, these results suggest that cyclotides like VarvA exert antimicrobial activity by disrupting bacterial membranes, along with previous reports. 

Biophysical studies have described that cyclotides target biological membranes [58]. Most cyclotides have conserved molecular surface regions, including hydrophobic and bioactive patches involved in the insertion into the membranes [59]. Several models for the interaction of AMPs with membranes, such as the “barrel stave,” “toroidal pore,” or “carpet” model, have been postulated [60]. In the carpet model, the peptides form a layer of “carpet” that induces membrane weakness, which ultimately results in membrane collapse by a detergent-like action. Moreover, the presence of membrane blebbing is associated with this model [61,62,63]. Thus, the SEM images support the notion that VarvA exerts a detergent-like mechanism to disrupt bacterial membranes. This idea is in agreement with the putative structure of the peptide, with hydrophobic patches able to interact with the membrane. Particularly, loop 6 composed of the first and the last amino acids form a cavity with the most hydrophobic residues on one side and the most hydrophilic on the other. This kind of hydrophobic distribution could favor the interaction with the bacterial membrane, first by the hydrophilic and charged residues to made the superficial contact, and second by the hydrophobic ones to interact with the lipid moiety of the membrane. Therefore, this study provides new evidence about the mechanism of action of cyclotides against the bacterial membrane.

## 4. Materials and Methods 

### 4.1. Peptide Synthesis

The cyclotide sequence of the VarvA cyclotide (cyclo(GLPVCGECFGGTCNTPGCSCDPWPMCSRN)) obtained from *Viola arvensis* [33] was chemically synthesized using the Fmoc-SPPS strategy. For this purpose, 2-chlorotrityl chloride (CTC) resin was loaded with 1.65 mmol of amino acid (Fmoc-Asn(Trt)-OH) per gram of resin and 5 eq. of *N*,*N*-diisopropylethylamine (DIEA) under anhydrous conditions with dicloromethane (DCM) as the solvent. The reaction mixture was stirred for 2 h. Then, 0.5 mL of methanol (MeOH) per g of resin was added and mixed for 5 min. The resin was then washed sequentially with DCM (×3), *N*,*N*-dimethylformamide (DMF) (×2) and DCM (×3), respectively, vacuum-dried, and weighed. 

The loading efficiency for the first amino acid was determined as previously described [64]. A sample of resin (5 mg) was placed in 2 mL microcentrifuge tubes (triplicate), and 1 mL 20% *v*/*v* piperidine in DMF was added to each tube. The tubes were vortexed briefly and allowed to agitate on a rotatory shaker (150 rpm) at room temperature for 20 min. Aliquots of 30 µL of each of the samples were diluted to 3 mL with DMF. The absorbance of each UV absorbance of each sample (300 nm) was determined against DMF as the reagent blank. Loading was calculated using the following equation: *Loading* = 101(A)/7.8(W), where A is the absorbance and W is the mass of the resin [64]. 

The cyclotide was synthesized on 40 mg resin (Fmoc-Asn(Trt)-O-CTC-resin) placed in polypropylene bags (74 μm mesh). The resin in these bags (quintupled) was swelled in a polypropylene bottle with DMF. The Fmoc group was removed by the addition of 20% *v*/*v* piperidine in DMF, for 10 min (twice). Next, the resin bags were washed 3 × 1 min in DMF, 1 × 1 min in isopropyl alcohol (IPA), 1 × 1 min in bromophenol blue (1% in DMF) for free amino group testing, and finally with 2 × 1 min with DMF and 2 × 1 min with DCM. The coupling reaction procedure was as follows: Fmoc-amino acid (10-fold excess) was activated by *N*-[(1H-benzotriazol-1-yl)-(dimethylamino) methylene]-*N*-methylmethanaminium hexafluorophosphate N-oxide (HBTU) in the presence of ethyl cyanohydroxyiminoacetate (OxymaPure^®^) and DIEA (5/5/5/7.5 milliequivalent (meq), respectively) in DMF. The coupling reaction was performed under vigorous shaking at room temperature for 3 h. The resin was then washed 2 × 1 min in DMF, and the second coupling reaction using TBTU as the activation reagent was performed immediately. Throughout the synthesis, DMF was used to wash the resin and to dissolve the amino acid, and all coupling and washing steps were performed under vigorous shaking. The cycle, starting with removal of the Fmoc group, was repeated until the last Fmoc-amino acid had been coupled. 

### 4.2. Cyclotide Cyclization and Oxidative Folding

Side chain protected linear peptides were first cleaved from the resin with 2% trifluoroacetic acid (TFA) in DCM for 30 min at room temperature (3 mL per bag). Next, 3 mL of water (Milli Q grade) was added, and TFA and DCM were then evaporated. The cyclization reaction was performed under highly diluted conditions in acetonitrile (ACN) with a peptide concentration of 0.5 mM. The cyclization reaction was carried out with 1% DIEA and 1 mM HBTU/Oxima and was stirred at room temperature for 1 h. After solvent evaporation, the peptide was dissolved in DCM, and sodium bicarbonate was added. The solution was centrifuged at 3000× *g* for 5 min. Sodium sulfate was then added and the mixture was centrifuged one more time. Finally, DCM was rota-evaporated.

Final deprotection was carried out with TFA/triisopropylsilane (TIS)/2,2′-(ethylenedioxy)-diethanethiol (DOTA)/water (Milli Q grade) (92.5/2.5/2.5/2.5 % *v*/*v*) at room temperature for 1 h. The crude peptides were precipitated and washed with cold diethyl ether. Finally, they were lyophilized and analyzed by MALDI-TOF mass spectrometry to confirm their molecular masses.

For the oxidative folding step, 10 mg of cyclic peptide was dissolved in water with 0.1 M ammonium bicarbonate (NH_4_HCO_3_) pH 8.0 [35]. The solution was stirred at room temperature for 1 h. Then aqueous solution was applied onto a Sep-pak C18 Vac cartridge (Waters Associates, Milford, MA, USA) equilibrated in acidified water (0.05% TFA) (Milli Q grade). After washing with acidified water (six times), the peptides were eluted at a flow rate of 1 mL/min with 5%, 10%, 20%, 30%, 40%, 60%, and 80% ACN in water. The appropriate fractions were collected, and the ACN was evaporated on a SpeedVac centrifuge. The fractions were then analyzed by reversed-phase (RP)-HPLC on a Water Corp XBridge™ BEH C18 column (100 mm × 4.6 mm, 3.5 µm) using a 0–70% ACN gradient, water containing 0.05% TFA as solvent A, and ACN containing 0.05% TFA as solvent B, at a flow rate of 1 mL/min for 8 min.

### 4.3. Prediction of Peptide Structure

The Basic Local Alignment Search Tool (BLAST) was used to determine the homology of the sequence in the Uniprot database, and a comparison with the sequences found was made by multiple alignment in Jalview [65]. The I-Tasser server [66] was used to determine the three-dimensional structure, and the model obtained was refined using UCSF Chimera (http://www.rbvi.ucsf.edu/chimera) [37]. Additionally, a hydrophobic surface was generated using a 1.4 angstrom probe and the Kyte & Dolittle hydrophobicity scale (used by default in Chimera).

### 4.4. Antibacterial Assay

Antibacterial activity was determined using the microplate assay, as previously described [67,68,69,70]. A range of peptide concentrations (1–50 μM) were mixed with 100 µL of an exponential phase bacterial culture of *Vibrio anguillarum*, *Vibrio ordalii*, *Flavobacterium psychrophilum*, *Aeromonas hydrophila*, and *Aeromonas salmonicida*. In addition, a phospholipase-A2-derived synthetic peptide variant was used as a positive control [39]. The test was performed at a starting OD of 0.001 at 620 nm in the following: tryptic soy broth (TSB) for *A. salmonicida*, and *A. hydrophila*; TSB containing 1.5% NaCl for *V. anguillarum* and *V. ordalii*; and Anacker and Ordal’s (AOAE) liquid medium for *F. psychrophilum* [70,71,72]. Absorbance was measured after 16 h of incubation. Minimum inhibitory concentrations (MIC) were defined as the lowest concentration of peptide that inhibited the visible growth of bacteria [73]. MICs were measured in quadruplicate.

### 4.5. SYTOX Green Bacteria Permeabilization Assay

The SYTOX Green uptake assay was performed according to a previously described procedure [74,75]. Cultures of exponentially-grown *E. coli* and *F. psychrophilum* were diluted in 10 mM sodium phosphate buffer (pH 7.2) to a cell density of 1 × 10^6^ CFU/mL. Then, aliquots of 90 μL of this cell culture were deposited in optics real time PCR tubes and 5 μL of the solution of VarvA synthetic cyclotide (MIC concentration) and 5 μL of 100 μM SYTOX Green were added to the wells, and then the tubes were placed in the thermocycler (Agilent Mx3000p qPCR System). The thermocycler program was performed using the SYBR green filter selected, 40 cycles of 30 s at 37 °C (*E. coli*) or 24 °C (*F. psychrophilum*) with a reading at the end of each cycle. Control experiments were performed under the same conditions without the addition of peptide. 

### 4.6. Scanning Electron Microscopy (SEM)

Aliquots of mid-log phase *A. salmonicida, V. ordalii*, and *F*. *psychrophilum* were harvested by centrifugation at 1000× *g* for 5 min. Cell pellets were washed twice with 10 mM saline phosphate buffer (PBS) and resuspended in the same buffer. The cell suspension was incubated at 24 °C for 20 min with the MIC concentration of the peptide. After incubation, the cells were centrifuged and washed three times at 1000× *g* for 5 min with PBS. Bacterial pellets were deposited on a glass coverslip in a Petri dish for 20 min and then fixed in 500 µL of 2.5% *v*/*v* glutaraldehyde in PBS. Subsequently, the bacterial samples were dehydrated with a graded ethanol series, critical-point dried, and coated with platinum-palladium to avoid charging in the microscope. Microscopic examination was performed using a Hitachi SU 3500 scanning electron microscope (Hitachi Ltd. Tokyo, Japan).

## 5. Conclusions

Fmoc solid phase peptide synthesis on 2-chlorotrityl chlorine (CTC) resin using the “tea-bag” approach was used to generate the VarvA cyclotide identified previously from *Viola arvensis*. The antimicrobial activity of this synthetic cyclotide was studied against *Vibrio anguillarum*, *Vibrio ordalii*, *Flavobacterium psychrophilum*, *Aeromonas hydrophila*, and *Aeromonas salmonicida*, being the highest against *Flavobacterium psychrophilum*. In addition, membrane blebbing on the bacterial surface was observed after exposure to VarvA, showing the ability of this cyclotide to disrupt the bacterial membrane. It is proposed that this compound exerts a carpet mechanism, a notion that is also consistent with the proposed structural model. Thus, cyclotides represent an interesting alternative to the use of antibiotics in the control of fish farming infections.

## Figures and Tables

**Table 1:** Values of minimal inhibitory concentration (MIC) for synthetic cyclotide on different Gram-negative bacterial fish pathogens.

Gram-Negative Bacterial Strain	MIC (μM)
*A. salmonicida*	22.5 ± 0.7
*A. hydrophila*	22.5 ± 0.3
*V. anguillarum*	30 ± 1.2
*V. ordalii*	30 ± 0.8
*F. psychrophilum*	12.5 ± 0.3
